# Outcomes of patients admitted with malignant small bowel obstruction: a subgroup multicentre observational cohort analysis

**DOI:** 10.1007/s00423-024-03436-3

**Published:** 2024-08-06

**Authors:** N. B. Hupfeld, J. Burcharth, T. K. Jensen, I. Lolle, L. B. J. Nielsen, M. A. Tolver, A. P. Skovsen, H. G. Smith

**Affiliations:** 1 Department of Surgery, Copenhagen University Hospital-North Zealand, Hilleroed, Denmark; 2 Department of Gastrointestinal and Hepatic Diseases, Copenhagen University Hospital-Herlev and Gentofte, Herlev, Denmark; 3 Department of Surgery, Copenhagen University Hospital-Amager and Hvidovre, Hvidovre, Denmark; 4 Digestive Disease Center, Copenhagen University Hospital-Bispebjerg and Frederiksberg, Copenhagen, Denmark; 5 Department of Surgery, Sjaelland University Hospital, Koege, Denmark; 6 Department of Surgery, Slagelse Hospital, Slagelse, Denmark

**Keywords:** Malignant small bowel obstruction, Palliative care, Operative vs. non-operative management, Overall survival

## Abstract

**Introduction and purpose of the study:**

Small bowel obstruction (SBO) accounts for a substantial proportion of emergency surgical admissions. Malignancy is a common cause of obstruction, either due to a primary tumour or intra-abdominal metastases. However, little is known regarding the current treatment or outcomes of patients with malignant SBO. This study aimed to characterise the treatment of malignant SBO and identify areas for potential improvement and compare overall survival of patients with malignant SBO to patients with non-malignant SBO.

**Materials and methods:**

This was a subgroup analysis of a multicentre observational study of patients admitted with SBO. Details regarding these patients’ diagnoses, treatments, and outcomes up to 1-year after admission were recorded. The primary outcome was overall survival in patients with malignant SBO.

**Results:**

A total of 316 patients with small bowel obstruction were included, of whom 33 (10.4%) had malignant SBO. Out of the 33 patients with malignant SBO, 20 patients (60.6%) were treated with palliative intent although only 7 patients were seen by a palliative team during admission. Nutritional assessments were performed on 12 patients, and 11 of these patients received parenteral nutrition. 23 patients underwent surgery, with the most common surgical interventions being loop ileostomies (9 patients) and gastrointestinal bypasses (9 patients). 4 patients underwent right hemicolectomies, with a primary anastomosis formed and 1 patient had a right hemicolectomy with a terminal ileostomy. Median survival was 114 days, and no difference was seen in survival between patients treated with or without palliative intent.

**Conclusion:**

Malignant SBO is associated with significant risks of short-term complications and a poor prognosis. Consideration should be given to the early involvement of senior decision-makers upon patient admission is essential for optimal management and setting expectation for a realistic outcome.

**Supplementary Information:**

The online version contains supplementary material available at 10.1007/s00423-024-03436-3.

## Introduction

Small bowel obstruction (SBO) is a common condition that accounts for a substantial proportion of acute surgical admissions [1]. Malignancy is the third most common cause of SBO, after intra-abdominal adhesions and hernias [2, 3]. Patients with malignant SBO represent a heterogenous group, ranging from those with a single point of obstruction due to a primary tumour to those with multiple levels of obstruction due to intra-abdominal metastases or carcinomatosis. The underlying pathology of malignant SBO also varies. While most often due to intra-abdominal or pelvic malignancies, malignant SBO may also be caused by metastases from extra-abdominal cancers, such as breast cancers [4, 5]. The diversity of the underlying pathology and its associated prognosis leads to further challenges in determining the optimal management in this patient group.

One major challenge in managing this patient group lies in defining what constitutes successful management. In patients with a single point of obstruction due to a primary tumour, surgery may be potentially curative. However, in patients with more extensive disease, the benefits of surgical intervention are less certain [6]. In some patients, it may be possible to relieve or bypass the obstruction in order to facilitate enteral nutrition. Identifying the patients for whom this is possible can be very challenging and, in some cases, may not be possible prior to an operation. In other patients, admission with malignant SBO may be a pre-terminal event, where success should be defined by the adequate palliation of symptoms [7].

Although there have been a considerable number of studies published on the treatment and outcomes of malignant SBO, the overwhelming majority are from retrospective single-centre series [6]. Here we present a subgroup analysis of an observational multicentre study investigating the short- and mid-term outcomes of patients admitted with malignant SBO of any cause, addressing different management strategies (operative vs. non-operative and palliative vs. non-palliative management) comparing short- and mid-term outcomes of patients with malignant and non-malignant SBO in relation to morbidity and overall survival.

## Methods

The DASBO (Danish Audit of Small Bowel Obstruction) study was a multicentre observational cohort study that included consecutive patients admitted to six acute hospitals in Zealand, the most populous island in Denmark with approximately 2.3 million inhabitants. Each centre has adopted a standardized acute care bundle tailored for acute high-risk surgical conditions (bowel obstruction, perforation, ischaemia or haemorrhage), involving expedited diagnostics, early initiation of antibiotics, prompt surgical intervention when indicated, and postoperative extended close observation in a semi-intensive care unit or similar setting [8]. Patients aged ≥ 18 years with a radiological or clinical diagnosis of SBO between February 22nd, 2021, and June 18th, 2021, were eligible for inclusion. This included patients presenting with SBO due to right-sided colon cancers causing obstruction at the level of the ileocaecal valve but not those with left-sided colon cancers or large bowel obstruction alone, as the management of these patients differs substantially [9]. The study was registered on clinicaltrials.gov (NCT04750811) and approved by the Danish Data Protection Agency (P-2021-70), and consent was obtained from all participating patients. DASBO included patients with SBO of any cause, and the overall short-term and mid-term outcomes have already been reported [3, 10]. The current study is a post-hoc subgroup analysis of the original DASBO cohort focuses on the short- and mid-term outcomes of patients with malignant and non-malignant SBO and is reported according to STROBE guidelines [11].

Clinicopathological data were prospectively retrieved from electronic patient records and entered in a pseudo-anonymized format into a secure REDCap database [12] housed by The Capital Region of Denmark. In the current study, patients were followed up to the time of death or one year after original inclusion. Patients were stratified according to the cause of SBO (malignant versus non-malignant). Patients with malignant obstruction were further subdivided into those treated with or without palliative intent and those with localized or metastatic disease. Patients with missing data were excluded for the analyses related to those specific variables.

Follow-up was conducted in person during admission and using electronic patient records after the initial discharge. All hospitals on Zealand use Sundhedsplatformen^®^ (EPIC Systems, Verona, Wisconsin) electronic patient records, allowing readmissions in any of the hospitals in this region to be recorded and followed. Furthermore, electronic patient records in Denmark are automatically updated in the case of a patient’s death, allowing mortality rates to be calculated with certainty. Data was checked for completeness by the principal investigator (HGS) and validated by the local investigators for each centre (TKJ, IL, LBJN, APS, MAT).

This study’s primary endpoints were 1-year overall survival. Secondary endpoints included 30-day morbidity according to the Clavien-Dindo classification [13] (complications higher than Grade II were recorded) and 90-day mortality rates, the proportion of patients treated with palliative versus curative intent, the proportion of patients undergoing operative versus non-operative management and the success rate of non-operative management. Descriptive statistics comparing demographics between groups were performed using the Chi-square test for categorical data and the Kruskal-Wallis test for continuous data. All statistical comparisons were two-sided, and a p-value of < 0.05 was considered statistically significant. Overall survival was calculated using the Kaplan-Meier estimate and compared using the log-rank test. All analyses were performed using SPSS version 25.0 (IBM, Armonk, New York, USA).

## Results

**Fig. 1:**
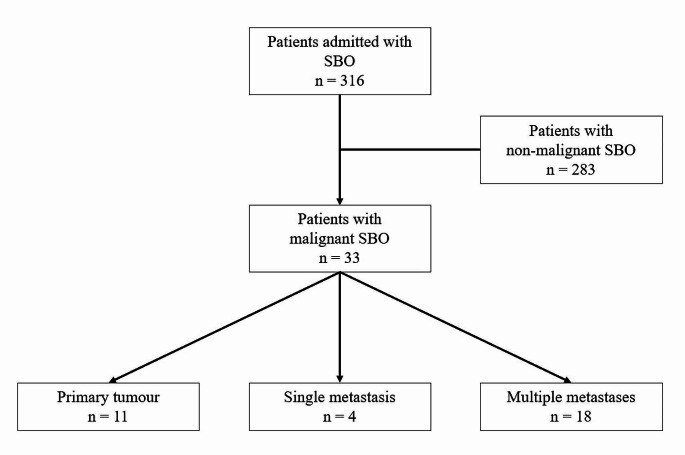
CONSORT diagram of the study cohort. SBO = small bowel obstruction

The original DASBO study included 316 patients [3], of whom 33 (10.4%) had malignant SBO (Fig. 1).

Of these, 18 patients (54.5%) had obstruction due to disseminated intra-abdominal malignancy, and four (12.1%) had obstruction due to a single isolated intra-abdominal metastasis. A summary of the underlying pathology in these patients is shown in Supplementary Table 1. No patients were lost to follow-up.

The remaining 11 patients (33.3%) had obstruction due to a localized primary tumour. Right-sided colon cancers (*n* = 6, 54.5%), obstructing the outlet of the small bowel through the ileocecal valve, were the most common localized tumours, followed by neuroendocrine tumours of the small bowel (*n* = 4, 36,4%). The remaining patient had SBO due to a locally advanced pancreatic adenocarcinoma. The clinicopathological demographics of patients with malignant SBO, compared to non-malignant SBO, are shown in Table 1.

**Table 1:** Demographics of patients admitted with malignant or non-malignant SBO

		Type of obstruction		
		Malignant	Non-malignant	*P* value
Number of patients		33	283	-
Male: female		0.7	0.5	0.448
Median age in years (IQR)		72 (60–77)	72 (56–79)	0.956
ASA grade	1	3 (9.1)	37 (13.1)	0.105
	2	9 (27.3)	121 (42.8)	
	≥ 3	21 (63.6)	125 (44.2)	
WHO Performance status	0	11 (33.3)	137 (48.4)	0.007
	1	5 (15.2)	78 (27.6)	
	≥ 2	17 (51.5)	74 (26.1)	
	Missing	0 (0)	2 (0.7)	
Median CCI (IQR)		7 (4–9)	4 (2–5)	< 0.001
Patient type	New	27 (81.8)	248 (87.6)	0.347
	Inpatient	6 (18.2)	35 (12.4)	
Nutritional Risk Index	Low	8 (24.2)	122 (43.1)	0.012
	Moderate	12 (36.4)	103 (36.4)	
	High	10 (30.3)	36 (12.7)	
	Missing	3 (9.1)	22 (7.8)	
AKI on admission		7 (21.2)	58 (20.5)	0.923
Median WCC (IQR)		11.7 (9.4–14.8)	10.0 (7.7–13.3)	0.078
Median CRP (IQR)		32.0 (15.0-95.3)	9.0 (4.0–37.0)	< 0.001
Median lactate (IQR)		1.2 (0.8–2.2)	1.2 (0.8–1.7)	0.602

IQR = interquartile range, ASA = American Society of Anesthiologists, WHO = World Health Organisation, CCI = Charlson Comorbidity Index, AKI = Acute kidney injury, WCC = white cell count, CRP = C-reactive protein

Patients with malignant SBO had more comorbidities, worse WHO performance status, and were more likely to be at risk of malnutrition than those with non-malignant SBO. Median serum C-reactive protein (CRP) levels were also higher in patients with malignant SBO, but no differences in white cell count (WCC) or arterial lactate levels were noted. Active cancer treatment had been given within eight weeks prior to admission in eight patients with malignant SBO (five received chemotherapy, two received targeted therapy, and one was treated with immunotherapy).

### Diagnosis and treatment strategies

The diagnosis of SBO was made using computed tomography (CT) scanning in almost all patients in the DASBO study (*n* = 313, 99.1%) and no differences in the use of imaging modalities were noted between patients with malignant or non-malignant SBO. However, patients with malignant SBO were less likely to be initially managed with an acute care bundle (comprising urgent CT scanning, broad spectrum antibiotics, nasogastric decompression and intravenous resuscitation) when compared to patients with non-malignant SBO (19/33 (57.6%) versus 230/283 (81.3%), *p* = 0.002). None of the patients with malignant SBO had radiological suspicion of bowel ischaemia or perforation, with two (6.1%) showing clinical signs of peritonism.

A total of 16 patients with malignant SBO underwent acute operations as the initial treatment strategy (48.5%). The rate of acute operations was higher in those patients with a single intra-abdominal tumour or metastasis when compared to those with multiple metastases, although this was not statistically significant (70.0% versus 39.1%, *p* = 0.103). The remaining 17 patients underwent a trial of non-operative management, which was successful in ten patients (58.8%). As such, a total of 23 patients underwent surgery for malignant SBO, with the most common surgical interventions being loop ileostomies (9 patients) and gastrointestinal bypasses (9 patients). The remaining five patients underwent right hemicolectomies, with a primary anastomosis formed in four patients and a terminal ileostomy in one patient. Of the 18 patients undergoing loop-ileostomies or bypasses, seven were re-admitted with SBO, with a median time to recurrence of 95 days (range 2–222 days) While the proportion of patients undergoing a trial of non-operative management did not differ significantly when compared to those with non-malignant SBO (51.5% versus 47.7%, *p* = 0.716), the success rate of this strategy was significantly lower in patients with malignant SBO (58.8% versus 81.5%, *p* = 0.031). A tendency towards longer trials of non-operative management was seen in patients with malignant SBO, whereby 3 out of these 7 patients continued non-operative management for > 72 h before eventual surgery.

### Palliative care and nutrition

Treatment, either operative or non-operative was given with palliative intent in 20 patients (60.6%). However, only seven (35.0%) of these patients were seen by a member of a specialised palliative care team during their admission. A trial of non-operative management was performed in 11 of these patients, and while the majority were given water-soluble contrast (10 patients), only three patients received intravenous steroids. Non-operative management was successful in five (45.5%) of these patients, with the remaining six undergoing surgery.

Formal nutritional assessments were only performed on 12 (36.4%) of the 33 patients with malignant SBO and were performed after treatment had already begun in most cases (75%). Parenteral nutrition was given to 11 of the 12 patients (91.7%) and was continued for more than 72 h in most of the patients who received it (9 out of 11 patients, 81.8%). Re-establishment of normal oral intake was delayed in patients with malignant SBO when compared to those with non-malignant SBO, with a significantly higher proportion of patients going more than five days without enteral intake after initial admission (malignant SBO 33.3% versus non-malignant SBO 17.6%, *p* = 0.031).

### Outcomes

The short-term outcomes of patients with malignant and non-malignant SBO are summarized in Table 2. Severe complications were noted in 8 of the 33 patients with malignant SBO, the majority of which were medical (in 5 patients). Of the 3 patients developing surgical complications, 2 had recurrence of SBO and 1 had a full abdominal dehiscence.

**Table 2:** Short-term outcomes of patients with malignant and non-malignant small bowel obstruction

	Type of obstruction		
	Malignant	Non-malignant	*P* value
Number of patients	33	283	-
30-day morbidity	8 (24.2)	46 (16.3)	0.326
Median length of stay (IQR)	10 (6–21)	4 (2–9)	< 0.001
30-day mortality	5 (15.2)	18 (6.4)	0.077
90-day mortality	15 (45.5)	24 (8.5)	< 0.001

IQR = interquartile range

**Fig. 2:**
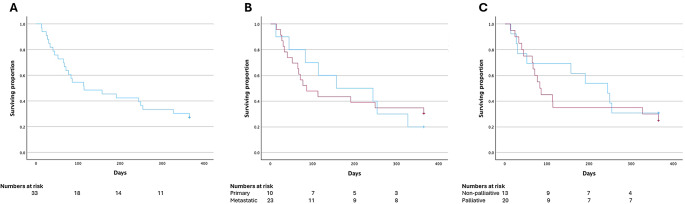
(**a**) Overall survival for all patients with malignant small bowel obstruction. (**b**) Overall survival in patients with primary (blue) or metastatic (red) disease. (**c**) Overall survival in patients treated with (red) or without (blue) palliative intent

No statistically significant differences in the rates of 30-day morbidity (*p* = 0.326) or mortality (*p* = 0.077) were noted between these groups consistent with findings from other studies [9]. However, patients with malignant SBO had a significantly longer length of hospital stay (median length of stay for malignant SBO of 10 days versus non-malignant of 4 days, *p* < 0.001) and significantly higher rates of 90-day mortality (malignant SBO 45.5% versus non-malignant SBO 8.5%, *p* < 0.001). Within one-year of the admission with malignant SBO, a total of 24 patients had died (72.7%). The median survival from admission with malignant SBO was 114 days (Fig. 2a). No significant difference in survival was seen between patients with malignant SBO due to a primary tumour or metastatic disease (log rank test, *p* = 0.993, Fig. 2b) or between those treated with or without palliative intent (log rank test, *p* = 0.851, Fig. 2c). Similarly, no significant difference in survival was seen between patients with malignant SBO treated operatively or non-operatively (log rank test, *p* = 0.922).

## Discussion

The management of patients with malignant SBO is challenging and most literature on this topic comprises small retrospective, and often single centre, series. The current study represents one of few observational multicentre subgroup analysis performed on this topic and highlights several important findings. The first is that malignancy is a relatively common cause of SBO. These patients accounted for just over 10% of all patients in the current cohort, with only adhesions and hernias being more common causes of SBO [3]. A similar frequency of malignant SBO has also been reported in other large cohort studies [2]. The second is that patients with malignant SBO represent a high-risk group for short term complications. Alongside non-statistically significant increases in 30-day morbidity and mortality, the rates of 90-day mortality in patients with malignant SBO in the current study were 5 times higher than in patients with non-malignant SBO. This is in keeping with previous prospective and retrospective cohort studies, where malignant SBO was found to be associated with increased risks of complications and in-hospital mortality compared to other causes of SBO [9, 14].

Perhaps more importantly, however, the current study also demonstrates that these patients have a poor prognosis even after their initial admission. Almost 3 of every 4 patients with malignant SBO in the current series had died within a year of their initial admission, and the cohort’s median survival was 114 days. Few studies have investigated the longer-term outcomes of patients with malignant SBO and those that have only included patients undergoing surgery [15, 16]. There is a risk of selection bias towards operating the fitter patients with less extensive metastatic disease, and therefore resulting in a better outcome, in this group. Somewhat surprisingly, in the current study, no difference was found in overall survival between patients with malignant SBO that were treated operatively or non-operatively. This suggest that the opposite could also be true – that the patients fairing worse are chosen for surgery as a non-operative approach is considered a non-viable option for the patient. This dilemma highlights the need for early and honest discussions with patients regarding their likely prognosis and what the primary goals of treatment should be, and the importance of making this discussion a priority as soon as the patient is admitted and diagnosed.

Given that admission with malignant SBO in many cases is a pre-terminal event, optimal palliation of the individual patient’s symptoms should be one of the key goals of treatment. Although guidelines for the treatment of malignant SBO have been developed [17], the diversity in terms of underlying pathology, viable oncological treatment options and life expectancy within this group means that the best palliative approach is likely to vary from patient to patient. Surgical intervention aiming to relieve or bypass the obstruction can form an important component of a palliative strategy. However, there is marked variation in the reported outcomes of palliative surgery, most of which come from small single centre studies. In a meta-analysis of 17 studies including 868 patients with malignant SBO due to peritoneal metastases, the rate of symptom resolution following palliative surgery ranged from 32 to 100% [18]. This potential benefit must be balanced against the risk of severe complications, which were seen in 7–44% of patients, as well as the risk of re-obstruction, which occurred in 38–47% of patients. Similar rates of complications and re-obstruction were seen in patients undergoing palliative surgery in the current study. A recent retrospective study of over 5,000 patients with malignant SBO found no difference in survival between those treated operatively or non-operatively [19]. However, those patients who underwent surgical intervention were less likely to be discharged to their own home and had fewer hospital-free days within 90 days of their initial admission. Given these risks, a non-operative treatment of malignant SBO is a potentially better palliative strategy, but the struggle of patient selection remains. Conversely for some patients offered a non-operative approach, a palliative surgery may improve the quality of life. Unfortunately, we do not have Quality of Life (QOL) data in our present cohort, which can be a valuable addition in future studies as it’s crucial for understanding the broader impact of the disease and its treatment on patients’ lives.

The current study has also highlighted some areas for potential improvement. Both inter- and intra-national variation in the management of patients admitted with SBO of any cause has been previously demonstrated [2, 20, 21]. Therefore, it is perhaps unsurprising that variations were also noted in the treatment of patients with malignant SBO. The use of acute care bundles is associated with reductions in mortality in patients with emergency abdominal presentations, including SBO [22], but these bundles were less commonly used in patients with malignant SBO. The reasons for this are unclear but it may represent the diagnostic difficulties of differentiating SBO from other symptoms related to the underlying malignancy or its treatment. Another potential concern is that only a minority of patients treated with palliative intent were seen by specialised palliative care teams during their admission. Access to palliative care services is known to vary, which can adversely impact the quality of end-of-life care [23]. In many centres in Denmark, end-of-life care is managed by the surgical team due to limited access to these services. However, a recent study has shown that nurse-led palliative services which are integrated within the surgical team can not only improve patient satisfaction but also help them focus on quality of life [24]. Expansion of similar services could be one potential strategy to improve the end-of-life care for this challenging group of patients.

Nutrition may be considered a further potential area for improvement. Formal nutritional assessments were performed in less than a third of patients in the current study, with a similar proportion of patients receiving parenteral nutrition. Similar rates of both nutritional assessments and therapy in this patient group have also been reported from other nations [25]. Malnutrition is common in patients with cancer, which can be further exacerbated by SBO, and has a recognised association with poorer survival outcomes [26, 27]. As such, one could argue that nutritional assessments and therapy should be a standard part of treatment in these patients. However, what is not clear is the extent to which malnutrition in patients with advanced malignancies, particularly those presenting with malignant SBO, is correctable rather than a representation of a general deterioration in a patient’s condition and prognosis. Correction of malnutrition during hospital admission appears particularly challenging in elderly patients with cancer [28]. Furthermore, the use of parenteral nutrition does not appear to impact survival in this setting [25]. As such, the decision to start parenteral nutrition requires careful consideration and honest discussion with patients regarding its potential benefits. Several criteria that should be considered are outlined in the current European Society for Parenteral and Enteral Nutrition guidelines and include the patient’s life expectancy and the likelihood that nutritional therapy will improve their quality of life [29].

## Limitations of the study

The authors recognise the limitations of this study. The number of patients with malignant SBO included is relatively low, and as such more extensive statistical analyses to identify prognostic factors of outcomes in this patient group were not feasible. In addition, the results of the current analyses should be interpreted with caution, given the study’s sample size. Furthermore, data regarding complications of Clavien Dindo grade ≤ 2 were not included. Whilst often regarded as minor, these complications can still lead to prolonged admissions, which may be particularly relevant in this patient group. In addition, although this was a multicentre study, only hospitals from eastern Denmark were included and, as such, the results may not be representative of national or international practice. The greatest limitation of the current study, and of the literature in general, is the lack of data regarding QOL in these patients. As described above, determining the optimal treatment of these patients can be incredibly challenging. Whilst arguments can be made for both operative and non-operative approaches, determining whether the right approach has been used in the right patient is nigh on impossible. Whilst the survival outcomes of patients treated operatively in the current study were no better than those treated non-operatively, it may well be that operations were performed on these patients because they were technically feasible or unavoidable. Larger prospective studies focusing on the quality of life in patients with malignant SBO, both at the time of admission and after eventual discharge, would be of great value in this difficult-to-treat patient group.

## Conclusion

In conclusion, malignant SBO is a common acute surgical presentation associated with high risks of short-term complications and a generally poor prognosis. Consideration should be given to the early involvement of senior decision makers during the admission of these patients to determine the most appropriate course of action, focusing on what outcomes are most relevant to the individual patient.

### Electronic supplementary material

Below is the link to the electronic supplementary material.

Supplementary Material 1

## Data Availability

No datasets were generated or analysed during the current study.

## References

[1] Gale SC, Shafi S, Dombrovskiy VY, Arumugam D, Crystal JS (2014) The public health burden of emergency general surgery in the United States: a 10-year analysis of the Nationwide Inpatient Sample–2001 to 2010. J Trauma Acute Care Surg 77(2):202–208. 10.1097/TA.0000000000000362 25058242 10.1097/TA.0000000000000362

[2] Lee MJ, Sayers AE, Drake TM, Marriott PJ, Anderson ID, Bach SP et al (2019) National prospective cohort study of the burden of acute small bowel obstruction. BJS Open 3(3):354–366. 10.1002/bjs5.50136 31183452 10.1002/bjs5.50136 PMC6551410

[3] Olausson M, Aerenlund MP, Azzam M, Bjerke T, Burcharth JFH, Dibbern CB et al (2023) Management and short-term outcomes of patients with small bowel obstruction in Denmark: a multicentre prospective cohort study. Eur J Trauma Emerg Surg 49(2):1121–1130. 10.1007/s00068-022-02171-y 36357790 10.1007/s00068-022-02171-y PMC9648885

[4] Miller G, Boman J, Shrier I, Gordon PH (2000) Small-bowel obstruction secondary to malignant disease: an 11-year audit. Can J Surg 43(5):353–358 11045093 PMC3695141

[5] Tuca A, Roca R, Sala C, Porta J, Serrano G, Gonzalez-Barboteo J et al (2009) Efficacy of granisetron in the antiemetic control of nonsurgical intestinal obstruction in advanced cancer: a phase II clinical trial. J Pain Symptom Manage 37(2):259–270. 10.1016/j.jpainsymman.2008.01.014 18789638 10.1016/j.jpainsymman.2008.01.014

[6] Cousins SE, Tempest E, Feuer DJ (2016) Surgery for the resolution of symptoms in malignant bowel obstruction in advanced gynaecological and gastrointestinal cancer. Cochrane Database Syst Rev 2016(1):Cd002764. 10.1002/14651858.CD002764.pub2 26727399 10.1002/14651858.CD002764.pub2 PMC7101053

[7] Ripamonti C, Twycross R, Baines M, Bozzetti F, Capri S, De Conno F et al (2001) Clinical-practice recommendations for the management of bowel obstruction in patients with end-stage cancer. Support Care Cancer 9(4):223–233. 10.1007/s005200000198 11430417 10.1007/s005200000198

[8] Burcharth J, Abdulhady L, Danker J, Ekeloef S, Jørgensen T, Lauridsen H et al (2021) Implementation of a multidisciplinary perioperative protocol in major emergency abdominal surgery. Eur J Trauma Emerg Surg 47(2):467–477. 10.1007/s00068-019-01238-7 31628502 10.1007/s00068-019-01238-7

[9] National Audit of Small Bowel Obstruction C (2019) Outcomes following small bowel obstruction due to malignancy in the national audit of small bowel obstruction. Eur J Surg Oncol 45(12):2319–2324. 10.1016/j.ejso.2019.07.014 31378418 10.1016/j.ejso.2019.07.014

[10] Mortensen MR, Alouda M, Bond Z, Burcharth J, Finne KF, Jensen TK et al (2023) One-year outcomes following operative or non-operative management of adhesional small bowel obstruction. BJS Open 7(5). 10.1093/bjsopen/zrad103 10.1093/bjsopen/zrad103 PMC10576245 37837353

[11] von Elm E, Altman DG, Egger M, Pocock SJ, Gotzsche PC, Vandenbroucke JP et al (2008) The strengthening the reporting of Observational studies in Epidemiology (STROBE) statement: guidelines for reporting observational studies. J Clin Epidemiol 61(4):344–349. 10.1016/j.jclinepi.2007.11.008 18313558 10.1016/j.jclinepi.2007.11.008

[12] Harris PA, Taylor R, Thielke R, Payne J, Gonzalez N, Conde JG (2009) Research electronic data capture (REDCap)--a metadata-driven methodology and workflow process for providing translational research informatics support. J Biomed Inf 42(2):377–381. 10.1016/j.jbi.2008.08.010 10.1016/j.jbi.2008.08.010 PMC2700030 18929686

[13] Clavien PA, Barkun J, de Oliveira ML, Vauthey JN, Dindo D, Schulick RD et al (2009) The Clavien-Dindo classification of surgical complications: five-year experience. Ann Surg 250(2):187–196. 10.1097/SLA.0b013e3181b13ca2 19638912 10.1097/SLA.0b013e3181b13ca2

[14] Wancata LM, Abdelsattar ZM, Suwanabol PA, Campbell DA Jr., Hendren S (2017) Outcomes after surgery for Benign and Malignant Small Bowel obstruction. J Gastrointest Surg 21(2):363–371. 10.1007/s11605-016-3307-8 27783343 10.1007/s11605-016-3307-8 PMC5263174

[15] Blair SL, Chu DZ, Schwarz RE (2001) Outcome of palliative operations for malignant bowel obstruction in patients with peritoneal carcinomatosis from nongynecological cancer. Ann Surg Oncol 8(8):632–637. 10.1007/s10434-001-0632-1 11569777 10.1007/s10434-001-0632-1

[16] de Boer NL, Hagemans JAW, Schultze BTA, Brandt-Kerkhof ARM, Madsen EVE, Verhoef C et al (2019) Acute malignant obstruction in patients with peritoneal carcinomatosis: the role of palliative surgery. Eur J Surg Oncol 45(3):389–393. 10.1016/j.ejso.2018.12.015 30594405 10.1016/j.ejso.2018.12.015

[17] Madariaga A, Lau J, Ghoshal A, Dzierzanowski T, Larkin P, Sobocki J et al (2022) MASCC multidisciplinary evidence-based recommendations for the management of malignant bowel obstruction in advanced cancer. Support Care Cancer 30(6):4711–4728. 10.1007/s00520-022-06889-8 35274188 10.1007/s00520-022-06889-8 PMC9046338

[18] Paul Olson TJ, Pinkerton C, Brasel KJ, Schwarze ML (2014) Palliative surgery for malignant bowel obstruction from carcinomatosis: a systematic review. JAMA Surg 149(4):383–392. 10.1001/jamasurg.2013.4059 24477929 10.1001/jamasurg.2013.4059 PMC4030748

[19] Bateni SB, Gingrich AA, Stewart SL, Meyers FJ, Bold RJ, Canter RJ (2018) Hospital utilization and disposition among patients with malignant bowel obstruction: a population-based comparison of surgical to medical management. BMC Cancer 18(1):1166. 10.1186/s12885-018-5108-9 30477454 10.1186/s12885-018-5108-9 PMC6258444

[20] Behman R, Karanicolas PJ, Nathens A, Gomez D (2021) Hospital-level variation in the management and outcomes of patients with Adhesive small bowel obstruction: a Population-based analysis. Ann Surg 274(6):e1063–e70. 10.1097/SLA.0000000000003739 31850993 10.1097/SLA.0000000000003739

[21] Tolver MA, MP AE, Azzam M, Bjerke T, Burcharth J, Dibbern CB et al (2023) Inter-hospital variation in management of patients with small bowel obstruction in Denmark. Dan Med J;70(9) 37622641

[22] Tengberg LT, Bay-Nielsen M, Bisgaard T, Cihoric M, Lauritsen ML, Foss NB (2017) Multidisciplinary perioperative protocol in patients undergoing acute high-risk abdominal surgery. Br J Surg 104(4):463–471. 10.1002/bjs.10427 28112798 10.1002/bjs.10427

[23] Boddaert MS, Pereira C, Adema J, Vissers KCP, van der Linden YM, Raijmakers NJH et al (2022) Inappropriate end-of-life cancer care in a generalist and specialist palliative care model: a nationwide retrospective population-based observational study. BMJ Support Palliat Care 12(e1):e137–e45. 10.1136/bmjspcare-2020-002302 33355176 10.1136/bmjspcare-2020-002302 PMC9120402

[24] Gerhardt S, Leerhøy B, Jarlbaek L, Herling S (2023) Qualitative evaluation of a palliative care case management intervention for patients with incurable gastrointestinal cancer (PalMaGiC) in a hospital department. Eur J Oncol Nurs 66:102409. 10.1016/j.ejon.2023.102409 37742424 10.1016/j.ejon.2023.102409

[25] Patel PS, Fragkos K, Keane N, Wilkinson D, Johnson A, Chan D et al (2024) Nutritional care pathways in cancer patients with malignant bowel obstruction: a retrospective multi-centre study. Clin Nutr ESPEN 59:118–125. 10.1016/j.clnesp.2023.11.018 38220364 10.1016/j.clnesp.2023.11.018

[26] Muscaritoli M, Lucia S, Farcomeni A, Lorusso V, Saracino V, Barone C et al (2017) Prevalence of malnutrition in patients at first medical oncology visit: the PreMiO study. Oncotarget 8(45):79884–79896. 10.18632/oncotarget.20168 29108370 10.18632/oncotarget.20168 PMC5668103

[27] Pressoir M, Desne S, Berchery D, Rossignol G, Poiree B, Meslier M et al (2010) Prevalence, risk factors and clinical implications of malnutrition in French Comprehensive Cancer centres. Br J Cancer 102(6):966–971. 10.1038/sj.bjc.6605578 20160725 10.1038/sj.bjc.6605578 PMC2844030

[28] Paillaud E, Caillet P, Campillo B, Bories PN (2006) Increased risk of alteration of nutritional status in hospitalized elderly patients with advanced cancer. J Nutr Health Aging;10(2):91 – 5 16554939

[29] Arends J, Bachmann P, Baracos V, Barthelemy N, Bertz H, Bozzetti F et al (2017) ESPEN guidelines on nutrition in cancer patients. Clin Nutr 36(1):11–48. 10.1016/j.clnu.2016.07.015 27637832 10.1016/j.clnu.2016.07.015

