# Supplementary material for: Outcomes of patients admitted with malignant small bowel obstruction: a subgroup multicentre observational cohort analysis
Source: Langenbecks Arch Surg. 2024 Aug 6;409(1):239. doi: 10.1007/s00423-024-03436-3 (PMC11303426; doi:10.1007/s00423-024-03436-3)
Supplement: Supplementary file 1 — Supplementary Material 1 [file 423_2024_3436_MOESM1_ESM.docx]

**Supplementary Table 1.** Site of initial primary cancer in patients presenting with malignant small bowel obstruction due to metastases.

| **Pathology** | **Number of patients** |
| --- | --- |
| Colorectal adenocarcinoma | 7 |
| Ovarian adenocarcinoma | 5 |
| Signet cell adenocarcinoma | 2 |
| Neuroendocrine cancer | 2 |
| Invasive ductal carcinoma (breast) | 1 |
| Pancreatic ductal adenocarcinoma | 1 |
| Duodenal adenocarcinoma | 1 |
| Unknown primary | 3 |
